# Development and validation of a multivariate predictive model for rheumatoid arthritis mortality using a machine learning approach

**DOI:** 10.1038/s41598-017-10558-w

**Published:** 2017-08-31

**Authors:** José M. Lezcano-Valverde, Fernando Salazar, Leticia León, Esther Toledano, Juan A. Jover, Benjamín Fernandez-Gutierrez, Eduardo Soudah, Isidoro González-Álvaro, Lydia Abasolo, Luis Rodriguez-Rodriguez

**Affiliations:** 1 Rheumatology Department, Hospital Clínical San Carlos, and IdISSC, Madríd, Spain; 2 International Centre for Numerical Methods in Engineering (CIMNE), Madrid, Spain; 3 Rheumatology Department, Hospital Clínico Universitario de La Princesa, and IIS-IP, Madrid, Spain

## Abstract

We developed and independently validated a rheumatoid arthritis (RA) mortality prediction model using the machine learning method Random Survival Forests (RSF). Two independent cohorts from Madrid (Spain) were used: the Hospital Clínico San Carlos RA Cohort (HCSC-RAC; training; 1,461 patients), and the Hospital Universitario de La Princesa Early Arthritis Register Longitudinal study (PEARL; validation; 280 patients). Demographic and clinical-related variables collected during the first two years after disease diagnosis were used. 148 and 21 patients from HCSC-RAC and PEARL died during a median follow-up time of 4.3 and 5.0 years, respectively. Age at diagnosis, median erythrocyte sedimentation rate, and number of hospital admissions showed the higher predictive capacity. Prediction errors in the training and validation cohorts were 0.187 and 0.233, respectively. A survival tree identified five mortality risk groups using the predicted ensemble mortality. After 1 and 7 years of follow-up, time-dependent specificity and sensitivity in the validation cohort were 0.79–0.80 and 0.43–0.48, respectively, using the cut-off value dividing the two lower risk categories. Calibration curves showed overestimation of the mortality risk in the validation cohort. In conclusion, we were able to develop a clinical prediction model for RA mortality using RSF, providing evidence for further work on external validation.

## Introduction

Rheumatoid arthritis (RA) is a chronic, systemic, inflammatory disease, characterized by inflammatory arthritis and localized destruction of bone, cartilage, and periarticular structures. This condition is associated with an increased mortality risk and a reduced life expectancy of about 3 to 10 years compared with the general population^1–5^.

Several socio-demographic and clinical-related factors with a significant impact in RA mortality have been identified^4–11^, mostly through the use of traditional survival techniques, such as the Cox proportional hazards (CPH) model^12^. However, these models have several limitations, including their reliance on restrictive assumptions, such as proportional hazards, being often parametric, therefore having to model nonlinear effects and interactions, which increases the risk of over-fitting and diminishes the statistical power of the model^13, 14^, and lacking reliability in the presence of high rates of censoring^15^.

In order to overcome these limitations and to improve the predictive performance, machine learning methods/models have been developed. These methods are able to “learn” from experience (data) and create predictive and prognostic models with high accuracy, reliability, and efficiency^16^.

Among the several machine learning methods that have been developed, random survival forest (RSF) has been proposed as an alternative approach to traditional survival methods^13^. RSF is a non-parametric method that generates multiple decision trees using bootstrap samples from the original data. Based on the majority votes of the individual decision trees, it is able to predict the outcome of interest. When the primary outcome is survival (time to event), RSF produces a cumulative hazard function (CHF) from each individual decision tree that are averaged in an ensemble CHF. RSF has been used for the analysis of right-censored survival data in several human diseases, such as cancer and cardiovascular diseases^17, 18^.

The objective of our study was to develop and validate, both internally and externally, a RSF prediction model of mortality in RA patients based on demographic and clinical-related variables collected during the first two years after disease diagnosis.

## Results

### Cohort description

1,461 patients from the Hospital Clínico San Carlos RA cohort (HCSC-RAC) and 280 RA patients from the Hospital Universitario de La Princesa Early Arthritis Register Longitudinal (PEARL) study were included in this study. The former is a day-to-day clinical practice cohort that includes subjects that have received a clinical diagnosis of RA by their usual rheumatologist^5^. The latter is an early arthritis cohort including RA and undifferentiated arthritis patients^19^. Demographic and clinical characteristics are shown in Table 1. Median follow-up time starting two years after RA diagnosis was 4.3 years [interquartile range (IQR): 2.0–6.8; range: 1 day-10.7 years] for the HCSC-RAC, and 5.0 (2.1–8.1; range: 3 days-11.3 years) for the PEARL. During follow-up, 148 (10.1%; time of observation of 6,707.6 person-years), and 21 (7.5%; time of observation of 1,441.4 person-years) patients died, resulting in a mortality rate of 22.1 [18.8 to 25.9], and 14.6 [9.5–22.3] events per 1,000 patients-year, respectively.

**Table 1:** Demographic and clinical characteristics of the rheumatoid arthritis patients from the “Hospital Clínico San Carlos - Rheumatoid Arthritis Cohort” and from the “Hospital Universitario de La Princesa Early Arthritis Register Longitudinal” with more than 2 years of follow-up after disease diagnosis.

Variables	HCSC-RAC		PEARL	
	n = 1,461	Missing data, n (%)	n = 280	Missing data, n (%)
Women, n (%)	1,105 (75.6)	0	223 (79.6)	0
Age of RA diagnosis, median (IQR)	58.6 (45.2–72.0)	0	54.9 (45.3–67.6)	0
Elapsed time from RA symptoms onset to diagnosis, in years, median (IQR)	0.7 (0.3 to 3.5)	180 (12.3)	0.5 (0.3–0.7)	0
Presence of Rheumatoid Factor, n (%)	885 (61.5)	23 (1.6)	181 (64.6)	0
Presence of ACPA, n (%)	465 (44.9)	425 (29.1)	234 (83.9)	1 (0.4)
Nationality, n (%):		0		0
Spanish	1,160 (79.4)	—	232 (82.7)	—
South/Centre America, Caribbean	237 (16.2)	—	37 (13.2)	—
Other	64 (4.4)	—	11 (3.9)	—
Year of RA diagnosis, n (%):		0		0
2001–2005	614 (42.0)	—	107 (38.2)	—
2006–2011	847 (58.0)	—	128 (45.7)	—
2012–2014	0		45 (16.1)	—
Median HAQ in the first 2 years after RA diagnosis, median (IQR)	0.50 (0.19–1.10)	376 (25.7)	0.63 (0.25–1.00)	1 (0.4)
Median ESR in the first 2 years after RA diagnosis, median (IQR)	23 (14 to 36.5)	248 (17.0)	20 (13–30)	1 (0.4)
Any biological therapy in the first 2 years after RA diagnosis, n (%)	89 (6.1)	0	28 (10.0)	0
Hospital admissions in the first 2 years after RA diagnosis, n (%)		0		13 (4.6)
0	1,258 (86.1)	—	230 (86.1)	—
1	144 (9.9)	—	28 (10.5)	—
2	39 (2.7)	—	5 (1.87)	—
3	16 (1.1)	—	2 (0.8)	—
≥4	4 (0.28)	—	2 (0.8)	—
Inclusion period, calendar years	2001–2011	—	2001–2014	—

ACPA: Anti-citrullinated peptides antibodies, ESR: Erythrocyte sedimentation rate, HAQ: Health assessment questionnaire, IQR: Interquartile range, RA: Rheumatoid Arthritis.

The variables presence of anti citrullinated peptide antibodies presence (ACPA) and median Health Assessment Questionnaire (HAQ) value in the first 2 years after RA diagnosis were excluded from the analysis due to their high proportion of missing data in the HCSC-RAC (Table 1).

### Model Development

First, we assessed that the number of trees included in the models were enough to obtain the lowest possible prediction error rate for that model, a measure of its discrimination ability. As showed online in Supplementary Figs S1 and S2, the higher the number of trees, the lower the prediction error. Furthermore, the prediction error stabilized above 200 trees, approximately, regardless the splitting rule use to construct the model [log-rank (M_LR_) or log-rank score (M_LRS_)].

The parameters used for the development of the M_LR_ and M_LRS_ are shown in Table 2. We present the results of the model using log-rank as splitting rule (M_LR_), since it exhibited the lowest prediction error, and therefore, the highest discrimination ability. In addition, the integrated Brier score (IBS; a measure of the model’s accuracy) for the overall follow-up was also lower when the log-rank splitting rule was used (Table 2 and Supplementary Figure S3).

**Table 2:** Parameters and quality measures of two random survival forests models using the log-rank or the log-rank score splitting rules developed for the prediction of mortality in a cohort of rheumatoid arthritis patients (HCSC-RAC).

Model	Splitting rule	Minimum terminal node size, n	Terminal nodes, mean	Variables tried at each split, n	Prediction error, mean (SD)	1 year IBS, mean (SD)	2 years IBS, mean (SD)	5 years IBS, mean (SD)	7 years IBS, mean (SD)	Overall IBS, mean (SD)
M_LR_	Log-rank	3	131.7	3	0.187 (0.002)	0.003 (0.0001)	0.013 (0.0004)	0.070 (0.002)	0.128 (0.003)	0.150 (0.003)
M_LRS_	Log-rank score	3	228.04	3	0.209 (0.003)	0.003 (0.0001)	0.012 (0.001)	0.071 (0.002)	0.140 (0.004)	0.167 (0.004)

IBS: Integrated Brier Score; SD: Standard deviation.

Next, we assessed the classification of the variables according to their predictive ability (Table 3), in order to select those variables to be included in the final model. The most important predictor variable was the patient’s age at the time of RA diagnosis, followed by the median erythrocyte sedimentation rate (ESR) during the first 2 years after RA diagnosis, the number of hospital admissions, the calendar year of RA diagnosis, and being Spaniard. The variables presence of rheumatoid factor (FR), use of any biological therapies during the first 2 years after RA diagnosis, elapsed time from RA symptoms onset to diagnosis, and gender also showed some predictive capacity, although considerably lower. Because all the variables showed a positive variable importance (VIMP), none was excluded from the final model, and therefore the M_LR_ was our final model.

**Table 3:** Variables included in the random survival forest M_LR_ ranked based on their variable importance value (VIMP).

Variables	VIMP, mean (SD)	IR (%)
Age of RA diagnosis	0.110 (0.001)	100
Median ESR in the first 2 years after RA diagnosis	0.014 (9.8 × 10^−4^)	12.7
Hospital admissions in the first 2 years after RA diagnosis	0.012 (7.0 × 10^−4^)	10.5
Calendar year of RA diagnosis	0.009 (9.1 × 10^−4^)	8.4
Spaniard	0.005 (5.3 × 10^−4^)	4.5
Presence of Rheumatoid Factor	6.1 × 10^−4^ (5.7 × 10^−4^)	0.6
Any biological therapy in the first 2 years after RA diagnosis	3.5 × 10^−4^ (1.7 × 10^−4^)	0.3
Elapsed time from RA symptoms onset to diagnosis	2.8 × 10^−4^ (9.4 × 10^−4^)	0.2
Gender	0.5 × 10^−4^ (5.5 × 10^−4^)	0.1

ACPA: Anti-citrullinated peptides antibodies; ESR: Erythrocyte sedimentation rate; HAQ: Health assessment questionnaire; RA: Rheumatoid Arthritis; SD: standard deviation; VIMP: Variable importance.

The effect on survival of these main variables was displayed with partial survival plots (Supplementary Figure S4) representing the predicted mortality rate for a given variable, after adjusting for all other variables. Older age at RA diagnosis, higher number of hospital admissions, higher median value of ESR during the first 2 years after RA diagnosis, and higher elapsed time from symptoms onset to diagnosis were associated with higher predicted mortality. Conversely, a more recent calendar year of RA diagnosis and the use of biological therapies were associated with lower mortality. In addition, we observed that the effect of the continuous variables in survival was not linear.

### Model Validation

After we developed our model using the HCSC-RAC, we used the data from the PEARL study to externally validate its performance. Using the M_LR_, we observed a prediction error in the validation cohort of 0.233. Comparing with the training cohort, we observed an increase in the prediction error, and therefore a worsening of the discrimination ability our model.

Finally, we performed a survival tree analysis using the individual predicted ensemble mortality from the HCSC-RAC to identify different mortality risk groups. The predicted ensemble mortality is the mean CHF estimated by the M_LR_ for each subject, and it was used as a measure of each patient estimated mortality risk. The cut-off values defining the risk groups are shown online in the Supplementary Table S1 and were applied to both cohorts. The mortality rate for each risk group and their comparison between groups, performed using a CPH model, both for the HCSC-RAC and for the PEARL study, are showed online in Supplementary Tables S2, and S3, respectively.

**Figure 1:**
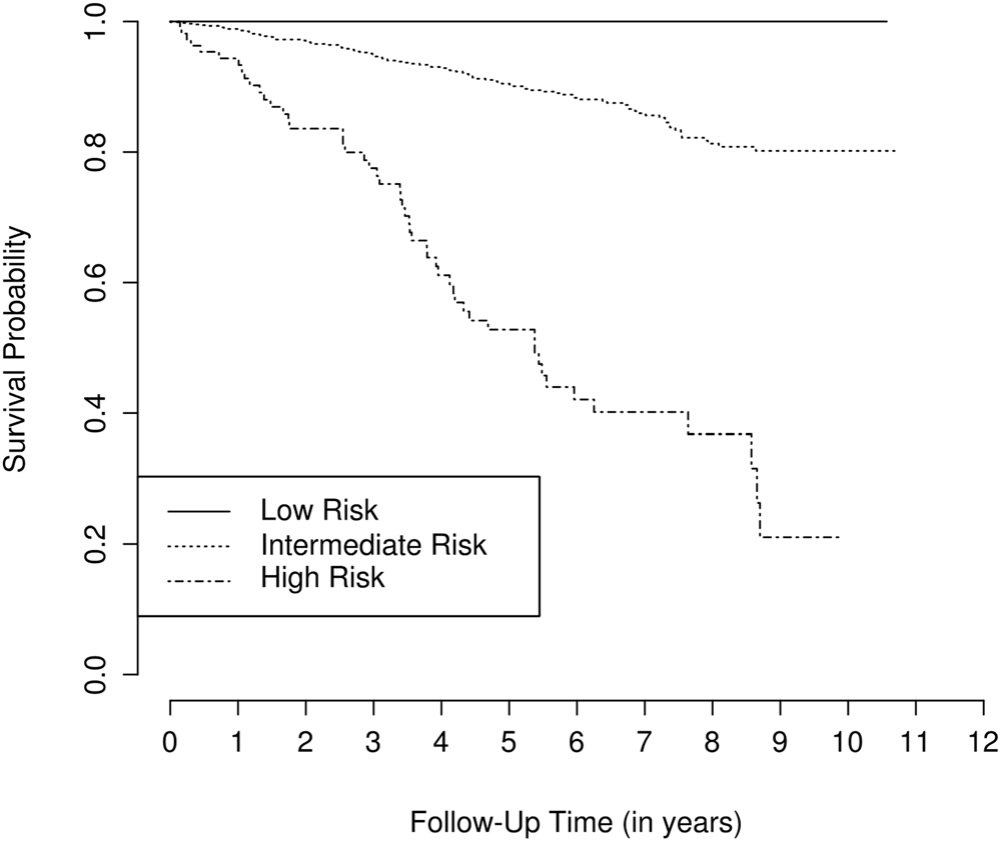
Kaplan Meier curves for the observed mortality of patients from the HCSC-RAC. Patient were grouped in mortality risk categories (low, intermediate, and high) according to a rheumatoid arthritis mortality random survival forest model using the log-rank splitting rule (M_LR_).

**Figure 2:**
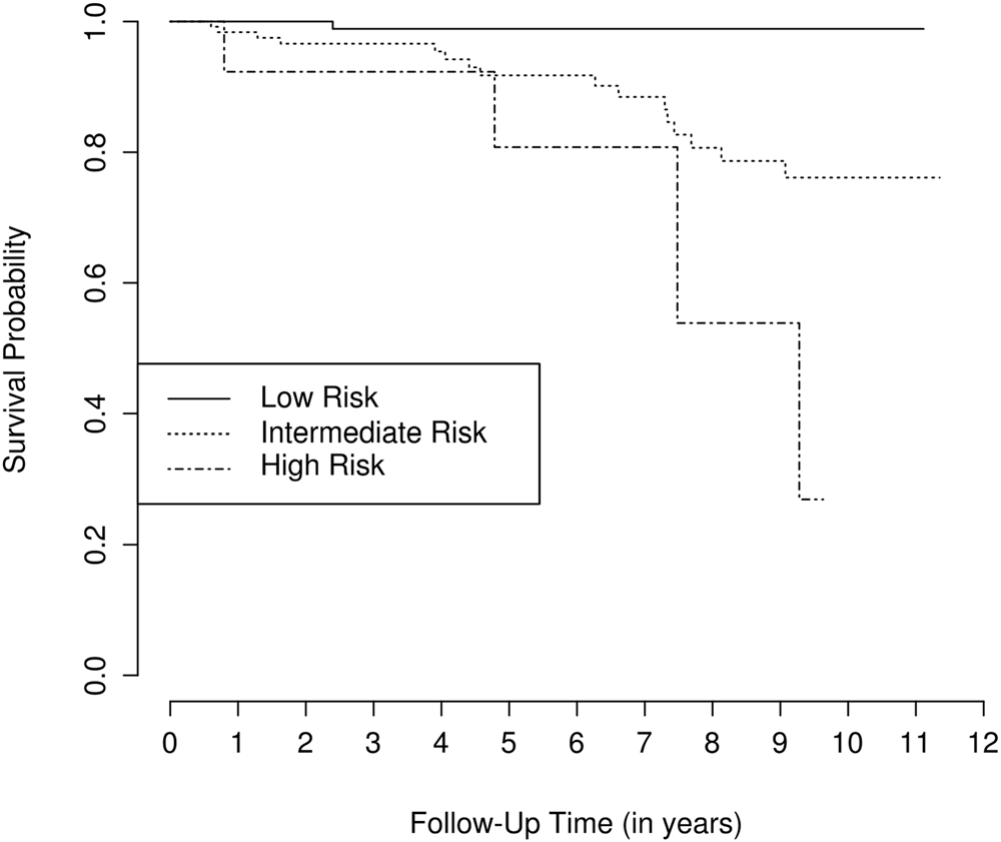
Kaplan Meier curves for the observed mortality of patients from the PEARL. Patient were grouped in mortality risk categories (low, intermediate, and high) according to a rheumatoid arthritis mortality random survival forest model using the log-rank splitting rule (M_LR_).

In order to reduce the number of groups and to maximize the differences among them, we decided to combine the three with intermediate risk, resulting in three final groups with low, intermediate, and high mortality risk (see Figs 1, 2, and Supplementary Table S4). In new CPH models, we observed that the intermediate and the high risk groups were significantly associated with higher mortality risk compared with the low risk, both in the training and in the validation cohorts.

As an example of how to apply the final model we developed and validated, we plotted the predicted survival curves of two different fictitious RA patients with different demographic and clinical characteristics (Supplementary Table S5 and Supplementary Figure S5). The predicted ensemble mortality were 1.1 and 12.5 for Patient A and B, respectively.

### Sensitivity, specificity, and model calibration

The time-dependent sensitivity and specificity was estimated for particular time points (1, 2, 5 and 7 years of follow-up, starting two years after RA diagnosis). Supplementary Figures S6 to S9 online show the relationship between sensitivity/specificity and the predicted individual ensemble mortality for that particular time point, both for the HCSC-RAC and the PEARL study. As expected, the higher the value of the predictive ensemble mortality, the higher the specificity and the lower the sensitivity. When compared both cohorts and regardless the time point and the predicted individual ensemble mortality, a greater specificity and a lower sensitivity was observed in the PEARL study. In the same way, similar results were observed for each of the cut-off points estimated using the survival tree analysis (online Supplementary Table S6).

Calibration curves for particular time points of follow-up (starting two years after RA diagnosis) are showed online in Supplementary Figures S10 and S11. For the HCSC-RAC, at 2 years of follow-up, the M_LR_ tended to underestimate the mortality risk of those with lower risk. Conversely, in those groups with higher risk, the model tended to overestimate the risk. For 5 and 7 years of follow-up, the model overestimate the risk for those groups with lower risk, and underestimate the mortality risk of those with higher risk. In the patients for the PEARL study, the M_LR_ tended to overestimate the mortality risk.

### Sensitivity Analysis

We developed two new models in the training cohort, constructed with the log-rank splitting rule (M_LR_): a) an “expanded model” (M_LRexp_), including all available variables, even those previously excluded due to their high proportion of missing values, and b) a “reduced model” (M_LRred_), including only those variables with greater predictive capacity. Both M_LRexp_ and M_LRred_ were compared with the final model, in terms of prediction error, IBS, and VIMP. We observed that our final model (M_LR_) had both a prediction error and an IBS between the expanded M_LR_ (M_LRexp_) and the reduced M_LR_ (M_LRred_; see Supplementary Table S7 online). Regarding the predictive capacity of the variables included in the M_LRexp_ (see Supplementary Table S8 online), both ACPA presence and median HAQ had a negative value, and their presence did not alter the ranking of the variables with higher predictive capacity. Regarding the M_LRred_, the same ranking as in M_LR_ was observed.

## Discussion

We have developed and externally validated a clinical predictive model for RA mortality using RSF, based on data collected during the first two years after disease diagnosis. To the best of our knowledge, this is the first time that this machine learning method is applied to analyze RA mortality. Furthermore, we identified several prognostic categories that were able to discriminate among subjects with different mortality risk in our validation cohort.

Several studies have used the RSF method to predict numerous disease outcomes, such as cancer mortality^17^ (including glioblastoma^20, 21^, leukaemia^22–25^, colon^26^, and thyroid^27^ cancer), cancer recurrence^28^, survival of kidney graft^15^, development of Huntington disease^29^, bed occupancy in an intensive care unit^30^, or time to initiation of symptomatic therapy in early Parkinson’s disease^31^. In rheumatology, RSF has been used to analyze the mortality risk in juvenile idiopathic inflammatory myopathies^32^, and in systemic lupus erythematosus^33^. Regarding the former, RSF was used to identify the most important variables of mortality in a cohort of 441 patients^32^. Later, those variables were included in a multivariate CPH model, with a prediction error of 23.4%. In addition, 3,839 SLE patients were used to create a model to predict in-hospital mortality, and to identify the most important variables. The prediction error of the best model was 11.9%. Regarding RA, although two studies used RSF to generate propensity scores to analyze, with a CPH model, the influence of methotrexate^34^ and corticosteroids^35^ in mortality, this method have not been used to predict mortality. Conversely, CPH models have been used in several studies to analyze the role of demographic and clinical-related variables in the mortality risk of RA patients. Although these studies uncovered numerous associations, they did not characterize these variables in terms of their capacity to predict mortality. Most of the variables that our model identified as having high predicting capacity have been associated with a higher mortality risk in most studies, including older age at inclusion^4, 5, 7, 9^ and higher disease activity^4, 7, 8^. In turn, among those variables with low predictive capacity, association studies have observed either conflicting results or lack of association: use of biological therapy has been associated with lower mortality in some, but not all studies^11, 36, 37^, probably due to the issue of confounding by indication^38^. Regarding the elapsed time from RA symptoms to diagnosis, Naz *et al*.^6^ observed lower mortality risk with greater elapsed time in a cohort of recent-onset inflammatory polyarthritis, although association was lost after adjustment.

However, we also observed contradictory results regarding our observations and previous studies. Some variables with high predictive capacity in our model lacked association with mortality in previous studies, such as calendar time. Most studies have observed either no clear influence of this variable in the mortality rate^4, 39^, or an small association with decreased risk^40^. In our study it was the fourth variable with the highest predictive capacity, even when considering that the effect of the other variables in the predictive capacity of calendar time in mortality was taken into account by the model. In the same way, variables with low predictive capacity in our model have showed a consistent association with mortality in most previous studies, including male gender^4, 6, 9^, or presence of RF^4, 6, 8, 9^.

Regarding hospital admissions, its total number during follow-up has been independently associated with a higher mortality risk in our RA cohort^5^. In turn, the number of the rate of hospitalizations has been associated with increased disease activity, and reduced physical activity^41, 42^. Therefore, we could consider this variable as a surrogate marker for RA severity. The fact that it showed predictive capacity even when another measure of disease activity (median ESR) was included in the model could mean that this variable conveys different aspects of the disease inflammatory burden, or other factors associated with mortality.

In daily practice, our prediction model could be implemented in an electronic health record. That way, the data needed for the model could be included automatically from the databases storing the information previously collected by physician or nurses during patient’s visits, or from the laboratories. In addition, the information from the model would be displayed in the same application used by the physician during the outpatient visits, not needing to access a different one. Considering the heavy workload of outpatient clinics that would facilitate the visualization of the results and its incorporation in the physician’s decision-making.

Our study has several limitations. Although we compared both splitting rules to produce the model with the lowest average prediction error, we used the default values for the rest of the parameters of the R package. In further studies, a fine tuning could increase the accuracy and precision of the models. In addition, despite we were able to develop a clinical model that showed accuracy and precision, we included a limited number of variables in our analysis. The inclusion of other variables, such as comorbilidy, lifestyle habits, prescribed treatments, or other potentially relevant variables, and the introduction of interaction among them, could improve the model. Furthermore, another limitation is the high proportion of missing data, particularly for disease activity, disability, and presence of ACPA in the HCSC-RAC. We want to point out that this is an observational study, using retrospective information from a cohort set in real life clinical practice, meaning that the data used was collected by physicians and nurses during day-to-day practice in an environment of heavy workload. Under those circumstances, higher rates of missing information are expected. In addition, there was no way to retrieve the missing information, as it was not collected (on paper or electronically) at the time of the patient’s appointment, or it was the result of erroneous or unsolicited laboratory tests. In addition, in our sensitivity analysis we observed that including those variables with higher proportion of missing data after imputation did not increase the predicting capacity of our models or modified the ranking of the predictive capacity of those variables included in the final model.

Another limitation is that, because RSF models involve thousands of decision trees collectively yielding a prediction, it is not possible to make a straightforward presentation of the classification criteria. However, it is a deterministic system, as the same combination of demographic and clinical characteristics will make the same prediction.

It is important to consider that the determination of the VIMP tends to favour continuous variables over categorical^43^. This could explain the low predictive capacity attributed to gender. However, another dichotomic variable, such as being Spaniard, ranked among those with higher predictive capacity. Therefore we think that this issue was not the main responsible for the low predictive capacity of this variable.

Finally, the same predicted ensemble mortality cut-off value showed a lower sensitivity in our external validation cohort. Considering that our outcome is mortality, it is important to take into account that the cost of a decision that results in a false negative (to consider a patient with high mortality risk as having low risk) is not the same as the cost of a false positive (to consider a patient with low mortality risk as having high risk), and that the former it is likely more desirable. In order to determine a satisfactory cut-off value we will need to test out model in new RA patient cohorts.

This study also has important strengths. We have used an independent RA cohort for external validation. Although most studies only perform an internal validation in order to assess the goodness-of-fit of the developed models, we also tested our model in an independent cohort, observing only a small increase in the prediction error. Comparing with previous studies of development and validation of prediction models, in a recent review^44^ the median C-index of samples used for external validation was 0.78 (IQR: 0.69–0.88), similar to what was observed in our study (C-index = 1 − prediction error = 0.77).

Regarding RA mortality prediction models, Provan *et al*.^45^ observed a C-index of 0.91 in multivariate logistic regression model of 10 year mortality including age, gender, disease activity and the value of the N-terminal pro-brain natriuretic peptide. Although their model exhibited higher discrimination ability, we want to point out that no internal or external validation was performed, and therefore we have to be cautious with the interpretation of their results. Morisset *et al*.^46^ developed a mortality model for patients with rheumatoid arthritis-associated interstitial lung disease. The cross-validated C-index of the final model (a multivariate CPH model) was 0.75, similar to our results. No external validation was performed by the authors.

It is important to point out that the number of events in that cohort was low (21 cases) and that some authors recommend to include at least 100–200 cases^47, 48^. Therefore, our model needs to be validated in different RA cohorts in order to properly evaluate its performance. In addition, it is important to point out that the same researchers that developed the prediction model carried out the external validation analysis, which could lead to bias^49^.

Another strength is that RSF methods do not rely on a restrictive assumption as traditional CPH models do, therefore requiring minimal data assumptions and automatically accounting for complex relationships among variables and with time^13, 31, 50–52^. Furthermore, RSF allows us to compare intuitively the predictive capacity among variables, adjusting for potential multiple interactions^13^. It also presents a reliable method for variable selection despite the presence of multicollinearity^13^, and it is able to reduce over-fitting due to the bootstrapping process used in the generation of decision trees. In line with this advantages over other methods, several studies have shown a higher generalization of the RSF results^18, 53–56^.

Finally, the partial plots have allowed us to visualize nonlinear relationships between variables and mortality, enabling us to identify cut-off point for these variables in further studies.

In conclusion, we have identified potentially modifiable mortality risk factors, which are mostly related to the inflammatory burden of RA. Therefore, a thorough control of inflammation during the early stages of disease could allow the patients to start out, after two years of RA diagnosis, with a lower mortality risk. In addition, we have developed a model that allows us, two years after RA diagnosis, to identify a subgroup of subjects with a higher mortality risk. Further studies need to be performed in order to assess if in this subgroup of patients a particular intervention can be implemented in order to reduce their risk.

## Methods

### Subjects

We performed a retrospective longitudinal study, using two independent RA cohorts to train and to externally validate our predictive model. For the training part of our study, we used the HCSC-RAC (Madrid, Spain). A thorough description of the cohort, including inclusion and exclusion criteria, follow-up and clinical assessments can be found in the article of Abásolo *et al*.^5^. Briefly, this is a day-to-day clinical practice cohort that includes subjects that have received a clinical diagnosis of RA by their usual rheumatologist. In this cohort we have included those patients that a) are attending or have attended the rheumatology outpatient clinic of the *Hospital Clínico San Carlos* (Madrid, Spain), with at least two registered visits, b) have received any ICD9 and/or ICD10 codes for RA by their usual rheumatologist, at least in two consecutive visits, c) were 16 years old or older at symptoms onset, and d) RA diagnosis was established from January 1, 1994 to February 15, 2013. In the case that the patient also receives a diagnosis of other autoimmune disease (such as inflammatory bowel disease, psoriasis or psoriatic arthritis, systemic lupus erythematosus, scleroderma, juvenile idiopathic arthritis, or ankylosing spondylitis), either before being diagnosed of RA or after being included in the RA cohort, his/her clinical record is reviewed by a rheumatologist (LA or LRR) that decides if the patient is included or excluded from the cohort, based on clinical, laboratory and treatment data. In addition to their routine clinical visits, patients included in the HCSC-RAC attend evaluation visits performed at baseline (when RA is diagnosed) and annually thereafter. In these visits, demographic, clinical and laboratory data is collected by a trained health professional evaluator. The present study was performed in a subset of the HCSC-RAC, selected based on a) the date of RA diagnosis and b) the length of follow-up. Because we wanted to minimize the missing information and to use data collected during the first two years after disease diagnosis, we included only those patients diagnosed in or after 2001, and those with at least 2 years of follow-up from the RA diagnosis.

For the external validation part of the study, we used the Hospital Universitario de La Princesa Early Arthritis Register Longitudinal (PEARL) study^19^. Briefly, this is an early arthritis cohort that includes patients diagnosed with RA^57^ or chronic undifferentiated arthritis^58^. Patient a) attending or that have attended the rheumatology outpatient clinic of the *Hospital Universitario La Princesa* (Madrid, Spain), and b) with 1 or more swollen joints at presentation, for at least 4 weeks and symptoms for less than a year, are included in the PEARL study. Patients are excluded if they are diagnosed with other specific cause of arthritis at presentation or during follow-up (such as gouty arthritis, septic or viral arthritis, osteoarthritis, spondyloarthropathies, or connective tissue diseases). Patients started to be included in PEARL since 2000. Patients attend 5 structured visits (at baseline, 6, 12, 24 and 60 months) in which sociodemographic, clinical, laboratory, therapeutic, radiological data and biological samples are systematically collected by protocol. In order to get more reliable data, especially regarding joint counts, these visits are performed by two experienced rheumatologist, but there is no pre-established therapeutic protocol, so the decision on when and how to treat the patients relies on the responsible physicians from the department. In the present study, only patients diagnosed with RA were included.

Informed consent was obtained from all the patients. This study was conducted in accordance with the Declaration of Helsinki and Good Clinical Practices^59^, and study protocols were approved by the HCSC and Hospital Universitario de La Princesa Ethics Committee.

### Variables

Our primary outcome was all cause mortality. For the HCSC-RAC, the date of death was obtained from the INDEF (*Índice Nacional de Defunciones*, Spanish for “National Mortality Index^60^), a national register depending on the Spanish Ministry of Health that records all deaths in the Spanish territory, regardless the nationality of the deceased (no cause of death is registered in this registry). The time of observation comprised the elapsed time between the date two years after the RA diagnosis and the date of patient’s death, or the date when mortality data was collected from the INDEF (September the 10, 2013).

For PEARL study, the date of death was obtained from HYGEIA (the electronic clinical tool used at Hospital Universitario La Princesa) or, in case of patients’ loss of follow-up, from HORUS (an electronic health record that integrates information collected from primary care centres, outpatient clinics, and hospital admissions in the Madrid Region). Therefore, the time of observation comprised the elapsed time between the date two years after the RA diagnosis and the date of patient’s death, loss of follow-up, or January the 1st, 2017).

Regarding independent variables, we used demographic and clinical-related variables, including gender (dichotomic), age and calendar year at RA diagnosis (continuous), nationality (dichotomic: Spaniard, not Spaniard), RF presence (dichotomic: yes, no), ACPA (dichotomic: yes, no), use of any biological therapy during the first two years after disease diagnosis (dichotomic: yes, no), number of hospital admissions regardless the cause during the first two years after disease diagnosis (continuous), and median values of the HAQ^61^ and of the ESR, performed at RA diagnosis, 1 and 2 years after (continuous)^8^. This information was obtained from a departmental electronic health record (Medi-LOG^62^), in the case of the HCSC-RAC, and from the PEARL database.

### Statistical analysis

Continuous variables were described using median and IQR, or mean and standard deviation (SD), based on their distribution. Dichotomous and categorical variables were described using proportions.

Random Survival Forests were implemented using the R software package *randomForestSRC* ^63^, developed by Ishwaran *et al*.^13^. Each run of RSF was performed based on 1,000 decision trees.

Regarding model training, the performance of the developed models was assessed using two measures: the prediction error^64^ and the IBS^65^. The prediction error is a measure of the model’s discrimination ability. It is equal to 1 – C-index^13^, which in turn is the probability that in two randomly selected pair of cases, the case with the shorter follow-up time has a worst predictive outcome^66–68^. The lower the prediction error (and therefore the higher the C-index), the better the model’s goodness of fit^13^. The IBS is a measure of the model’s accuracy, and it is calculated by squaring the differences between the patient primary outcome at a particular point in time (being alive or dead) and the predicted probability of this outcome at that time^69^. The lower the IBS, the better the model’s accuracy.

Those variables with more than 20% of missing data in the training cohort were excluded from the analysis (Table 1). For the rest of the variables, missing data was imputed using an iterative algorithm^13^ supplied by the *randomForestSRC* package. As showed in other studies^70^, this algorithm appears to be reliable.

Internal validation was performed though bootstrapping, meaning that each of the decision trees that integrate the forest are created from a subset (in-bag data) of a bootstrapped sample from the original dataset (in our case the patients from the HCSC-RAC). The other subset (out-of-bag data) of the bootstrapped sample is used to calculate the prediction error^71^.

Two RSF models were constructed applying either the log-rank^72, 73^ (M_LR_) or the log-rank score^74^(M_LRS_) splitting rules. In order to obtain an unbiased measure of the models quality, we performed 100 iterations of each model, and we estimated the mean and SD of the prediction error. We selected the model with the lowest prediction error, and ranked the included variables according to their predictive capacity by an internal measure of variable importance (VIMP). The VIMP compares a variable’s predictive power to its power under randomness. If the VIMP is close to zero, then the predictive capacity of that variable is lower, as the difference between the predictive power of the variable and its predictive power under randomness is small. Conversely, if the VIMP is large, then the predictive capacity of that variable is higher, as there is a greater difference between the predictive power of the variable and its predictive power under randomness. We calculated the mean (SD) VIMP based on 100 iterations of the model. In addition, we calculated for each variable their relative importance (RI), by dividing the mean VIMP score assigned to a specific variable by the mean VIMP score assigned to the first ranked variable. Based on the mean VIMP, we constructed a final model, excluding those variables with a negative VIMP^75^, and performed 100 iterations of the final model in order to estimate its prediction error, IBS, and VIMP of the included variables. We used partial plots to represent the effect on the predicted mortality of each variable included in the final model, after accounting for the average effects of the other variables.

Regarding external validation, the final model created with the patients from the HCSC-RAC was tested in a group of independent patients from the PEARL study, a different cohort of RA patients from a different centre, followed-up by different physicians, and collected and curated by different researchers. The observations from the PEARL cohort were dropped down the final RSF model and goodness-of-fit was assessed using the prediction error. Missing data was imputed using the iterative algorithm supplied by the *randomForestSRC* package^13^.

We also tested the performance of our final model by defining groups of patients based on their individual estimated mortality risks (i.e. groups with low, intermediate, and high risk) and then assessing and comparing the observed mortality among these groups. The out-of-bag mean cumulative hazard function^22^ (referred to as ensemble mortality) estimated by the final RSF model was used as a measure of each patient’s estimated mortality risk. For a particular patient, the ensemble mortality can be interpreted as the expected number of deaths in a cohort if all the patients had similar characteristics to those of this particular patient. The ensemble mortality cut-off values defining different risk groups were established using the data from the training cohort through a survival tree created with the R package *rpart* with default parameters^76^. These same cut-off values were used to define risk groups in the validation cohort. The survival probabilities along time for each mortality risk group were visually represented using Kaplan-Meier curves, and statistically compared with a bivariate Cox proportion hazard test, using the mortality risk group as a categorical variable and the lower risk category as reference. Differences in mortality risks among groups were expressed as hazard ratio (HR) and 95% confidence intervals (95% CI).

The relationship between time-dependent sensitivity/specificity and the ensemble mortality value for particular time points was estimated using the *survivalROC* R package^77^. Calibration was estimated using the *pec* R package^78^.

All analyses were performed by using R (version 3.3.2), the randomForestSRC, the *rpart*, the *timeROC*, and *survivalROC* the packages. A more detailed description of the statistical analysis can be found online in the Supplementary Methods).

### Sensitivity analysis

Two new models were developed in the training cohort and compared with the final model, in terms of prediction error, IBS, and VIMP:

- A model including the variables from the final model and those excluded due to their high percentage of missing values (expanded model).
- A model including the variables from the final model except those with positive but low predictive capacity (RI < 1%; reduced model).

### Data availability

The datasets analysed during the current study and the final Random Survival Forest Rheumatoid Arthritis Mortality Prediction Model are available from the corresponding author on reasonable request.

## Electronic supplementary material

Supplementary Information
