# Supplementary material for: Development and validation of a multivariate predictive model for rheumatoid arthritis mortality using a machine learning approach
Source: Sci Rep. 2017 Aug 31;7:10189. doi: 10.1038/s41598-017-10558-w (PMC5579234; doi:10.1038/s41598-017-10558-w)

SUPPLEMENTARY INFORMATION

Development and validation of a multivariate predictive model for rheumatoid arthritis mortality using a machine learning approach

José M Lezcano-Valverde, Fernando Salazar, Leticia León, Esther Toledano, Juan A. Jover, Benjamín Fernandez-Gutierrez, Eduardo Soudah, Isidoro González-Álvaro, Lydia Abasolo, Luis Rodriguez-Rodriguez.

Supplementary Methods

Random Survival Forest

This method generates multiple decision trees based on bootstrap data from the original sample, and predicts the outcome of interest based on the majority votes of the individual decision trees. Briefly, the algorithm can be described as follows:

1. Starting from the original data, n bootstrap samples are drawn. Each sample is randomly divided in two: in-bag data and out-of-bag data (OOB data).

2. From each bootstrap sample, a survival tree is grown using the in-bag data. At each node of the tree, a number of candidate variables (equal to the squared root number of candidate variables) are randomly selected, and the candidate variable that maximizes survival difference between the two daughter nodes is used for split the node.

3. The tree is grown until any of the daughter nodes have less that a certain number of events (in our case 3 deaths).

4. The ensemble cumulative hazard function (CHF) is obtained by averaging the CHF of each of the grown trees.

Statistical Analysis

The prediction error for the ensemble CHF was calculated by averaging the individual tree classification errors resulting from dropping the OOB observations down the decision tree created with the in-bag data. This is a measure of the discrimination ability of the model, and is equal to 1 – C-index (Harrell's concordance index[1–3]). The C-index is the probability that in two randomly selected pair of cases, the case with the shorter follow-up time has the worst predictive outcome[2,4,5]. It ranges from 0.5 (non-informative) to 1.0 (perfect discrimination). Conversely, for the prediction error, the lower, the better the prediction accuracy[1].

The Brier score was calculated by adding, at a particular point in time, the squared distance between a person's mortality status and their probability of being dead according to the model[6]. It ranges from 0 (perfect precision) to 1 (poor precision), being 0.25 non-informative[7,8].

Briefly, the iterative algorithm supplied by the *randomForestSRC* package[1] for imputation works as follows: previous to splitting each node in the step 2 of the algorithm, missing data for a variable is imputed with randomly selected values from the non-missing in-bag data. These imputed data are only used by the splitting but not for calculate the split-statistic, which is done with non-missing data only. After splitting, imputed data are reset to missing and the process is repeated until terminal nodes are reached. In terminal nodes, missing data are imputed using OOB non-missing terminal node data[9].

Briefly, *rpart* selects the mortality ensemble value that maximises differences in survival between daughter nodes at each split of the survival tree. This process continues until the number of subjects is too small to meaningfully split based on the mortality ensemble. The final nodes represent the risk categories defined by the cut-off values.

Supplementary References

1. Ishwaran, H., Kogalur, U. B., Blackstone, E. H. & Lauer, M. S. Random survival forests. *Ann. Appl. Stat.* **2,** 841–860 doi:10.1214/08-AOAS169 (2008).

2. Harrell, F. E., Califf, R. M., Pryor, D. B., Lee, K. L. & Rosati, R. A. Evaluating the yield of medical tests. *JAMA* **247,** 2543–6 doi:10.1001/jama.1982.03320430047030 (1982).

3. Penciana, M. J. & D'Agostino, R. B. Overall C as a measure of discrimination in survival analysis: Model specific population value and confidence interval estimation. *Stat. Med.* **23,** 2109–2123 doi:10.1002/sim.1802 (2004).

4. Heagerty, P. J. & Zheng, Y. Survival model predictive accuracy and ROC curves. *Biometrics* **61,** 92–105 doi:10.1111/j.0006-341X.2005.030814.x (2005).

5. Zweig, M. H. & Campbell, G. Receiver-operating characteristic (ROC) plots: A fundamental evaluation tool in clinical medicine. *Clin. Chem.* **39,** 561–577 doi:ROC; Receiver-Operating Characteristic; SDT; Signal Detection Theory (1993).

6. Mogensen, U. B., Ishwaran, H. & Gerds, T. A. Evaluating Random Forests for Survival Analysis Using Prediction Error Curves. *J. Stat. Softw.* **30,** 1–3 doi:10.1126/scisignal.2001449.Engineering (2009).

7. Simuni, T. *et al.* Predictors of time to initiation of symptomatic therapy in early Parkinson's disease. *Ann. Clin. Transl. Neurol.* 482–494 (2016).

8. Gerds, T. A. & Schumacher, M. Consistent estimation of the expected brier score in general survival models with right-censored event times. *Biometrical J.* **48,** 1029–1040 doi:10.1002/bimj.200610301 (2006).

9. Fontana, A. *et al.* Development of a metabolites risk score for one-year mortality risk prediction in pancreatic adenocarcinoma patients. *Oncotarget* **7,** 8968–8978 doi:10.18632/oncotarget.7108 (2016).

Table S1: Predicted mortality ensemble cut-off values defining mortality risk categories, obtained thought regression tree analysis.

Category Risk	Cut-off values
1	< 1.38
2	≥1.38, < 9.43
3	≥9.43, < 21.03
4	≥21.03, < 38.83
5	≥ 38.83

Table S2: Mortality rate of the Hospital Clínico San Carlos - Rheumatoid Arthritis Cohort based on five mortality risk score categories.

Category Risk	Person-time	Deaths	MR (95% CI)	HR (95% CI)	p-value
1	1,705.2	2	1.2 [0.3-4.7]	1.0	-
2	2,750.8	29	10.5 [73-15.2]	8.6 [2.1-36.1]	$3.2x10^{-3}$
3	1,212.3	32	26.4 [18.7-37.3]	21.6 [5.2-90.1]	$2.5x10^{-5}$
4	615.2	31	50.4 [35.4-71.7]	41.0 [9.8-171.3]	$3.6x10^{-7}$
5	424.0	54	127.3 [97.5-166.3]	108.8 [26.5-446.2]	$7.4x10^{-11}$

CI: Confidence interval; HR: Hazard ratio; MR: Mortality rate.

Table S3: Mortality rate of the Hospital Universitario de La Princesa Early Arthritis Register Longitudinal based on five mortality risk score categories.

Category Risk	Person-time	Deaths	MR (95% CI)	HR (95% CI)	p-value
1	646.5	1	1.5 [0.2-11.0]	1.0	-
2	485.6	6	12.4 [5.6-27.5]	7.6 [0.9-63.0]	0.061
3	178.6	7	39.2 [18.7-82.2]	26.6 [3.3-216.1]	$2.2x10^{-3}$
4	65.0	3	46.2 [14.9-143.2]	28.1 [2.9-270.8]	$3.9x10^{-3}$
5	65.8	4	60.8 [22.8-162.1]	41.0 [4.6-367.9]	$9.0x10^{-4}$

CI: Confidence interval; HR: Hazard ratio; MR: Mortality rate.

Table S4: Mortality rate of the Hospital Clínico San Carlos - Rheumatoid Arthritis Cohort and the Hospital Universitario de La Princesa Early Arthritis Register Longitudinal based on three mortality risk score categories

Category Risk	HCSC-RAC			PEARL		
	MR (95% CI)	HR (95% CI)	p-value	MR (95% CI)	HR (95% CI)	p-value
Low	1.2 [0.3-4.7]	1.0	-	1.5 [0.2-11.0]	1.0	-
Intermediate	20.1 [16.4-24.7]	16.4 [4.0-66.5]	$9.2x10^{-5}$	21.9 [13.4-35.8]	13.8 [1.8-104.4]	0.011
High	127.3 [97.5-166.3]	108.8 [26.5-446.2]	$7.4x10^{-11}$	60.8 [22.8-162.1]	41.0 [4.6-367.9]	$9.0x10^{-4}$

CI: Confidence interval; HR: Hazard ratio; MR: Mortality rate.

Table S5: Demographic and clinical-related characteristics of two fictional rheumatoid arthritis patients.

Variables	Patient A	Patient B
Age of RA diagnosis	65	69
Median ESR in the first 2 years after RA diagnosis	15	20
Hospital admissions in the first 2 years after RA diagnosis	0	2
Calendar year of RA diagnosis	2006	2008
Spaniard	Yes	No
Presence of Rheumatoid Factor	No	Yes
Any biological therapy in the first 2 years after RA diagnosis	No	Yes
Elapsed time from RA symptoms onset to diagnosis, years	0.5	1.5
Gender	Man	Woman

Table S6: Sensitivity and specificity for different time points during follow-up (starting two years after RA diagnosis) corresponding to the predicted mortality ensemble cut-off values defining mortality risk categories, obtained thought regression tree analysis.

	1 year		2 years		5 years		7 years	
Cut-off value	HCSC-RAC	PEARL	HCSC-RAC	PEARL	HCSC-RAC	PEARL	HCSC-RAC	PEARL
1.38 (Sensitivity/Specificity)	1.00 / 0.30	1.00 / 0.48	0.98 / 0.31	1.00 / 0.48	0.99 / 0.32	0.81 / 0.48	0.99 / 0.33	0.84 / 0.49
9.43 (Sensitivity/Specificity)	0.98 / 0.68	0.43 / 0.79	0.77 / 0.69	0.47 / 0.80	0.69 / 0.70	0.42 / 0.80	0.70 / 0.72	0.48 / 0.80
21.03 (Sensitivity/Specificity)	0.54 / 0.85	0.22 / 0.92	0.44 / 0.85	0.18 / 0.92	0.4 / 0.86	0.19 / 0.92	0.38 / 0.87	0.19 / 0.92
38.83 (Sensitivity/Specificity)	0.22 / 0.92	0.16 / 0.95	0.23 / 0.93	0.12 / 0.95	0.21 / 0.93	0.12 / 0.96	0.20 / 0.94	0.12 / 0.96

Table S7: Parameters and quality measures of two random survival forests models using the log-rank splitting rule including all available variables (expanded model: M_{LRexp}) of only those variables with a relative variable importance >1% (reduced model: M_{LRred}).

Model	Splitting rule	Minimum terminal node size, n	Terminal nodes, mean	Variables tried at each split, n	Prediction error, mean (SD)	1 year IBS, mean (SD)	2 years IBS, mean (SD)	5 years IBS, mean (SD)	7 years IBS, mean (SD)	Overall IBS, mean (SD)
M_{LRexp}	Log-rank	3	132.3	4	0.189 (0.007)	0.003 ($0.1x10^{-4}$)	0.012 ($0.5x10^{-4}$)	0.068 ($5.4x10^{-4}$)	0.122 (0.001)	0.143 (0.001)
M_{LRred}	Log-rank	3	125.1	3	0.181 (0.005)	0.003 ($1.0x10^{-4}$)	0.014 ($3.0x10^{-4}$)	0.077 (0.001)	0.139 (0.001)	0.165 (0.002)

IBS: Integrated Brier Score; SD: Standard deviation.

Table S8: Variables included in the random survival forest expanded (M_{LRexp}) and reduced (M_{LRred}) models ranked based on their variable importance value (VIMP).

Variables	M_{LRexp} VIMP, mean (SD)	M_{LRexp} IR (%)	M_{LRred} VIMP, mean (SD)	M_{LRred} IR (%)
Age of RA diagnosis	0.110 ($4.6x10^{-4}$)	100	0.122 ($5.1x10^{-4}$)	100
Median ESR in the first 2 years after RA diagnosis	0.012 ($3.3x10^{-4}$)	10.8	0.019 ($3.9x10^{-4}$)	15.4
Hospital admissions in the first 2 years after RA diagnosis	0.011 ($2.1x10^{-4}$)	10.3	0.016 ($1.9x10^{-4}$)	13.1
Calendar year of RA diagnosis	0.006 ($2.5x10^{-4}$)	5.2	0.010 ($2.8x10^{-4}$)	8.5
Spaniard	0.004 ($1.5x10^{-4}$)	3.4	0.001 ($1.1x10^{-4}$)	0.7
Gender	$7.2x10^{-4}$ ($1.7x10^{-4}$)	0.7	-	-
Presence of Rheumatoid Factor	$3.2x10^{-4}$ ($1.6x10^{-4}$)	0.3	-	-
Any biological therapy in the first 2 years after RA diagnosis	$1.4x10^{-4}$ ($0.5x10^{-4}$)	0.1	-	-
Elapsed time from RA symptoms onset to diagnosis	$-9.4x10^{-4}$ ($2.4x10^{-4}$)	-0.9	-	-
Presence of ACPA	-0.001 ($1.5x10^{-4}$)	-1.0	-	-
Median HAQ in the first 2 years after RA diagnosis	-0.002 ($2.3x10^{-4}$)	-2.0	-	-

ACPA: Anti-citrullinated peptides antibodies; ESR: Erythrocyte sedimentation rate; HAQ: Health assessment questionnaire; RA: Rheumatoid Arthritis; VIMP: Variables importance.

Figure S1: Effect that the number of decision trees included in a random survival forest (x-axis) has in the prediction error (y-axis) of a rheumatoid arthritis mortality model using the log-rank splitting rule (M_{LR}).

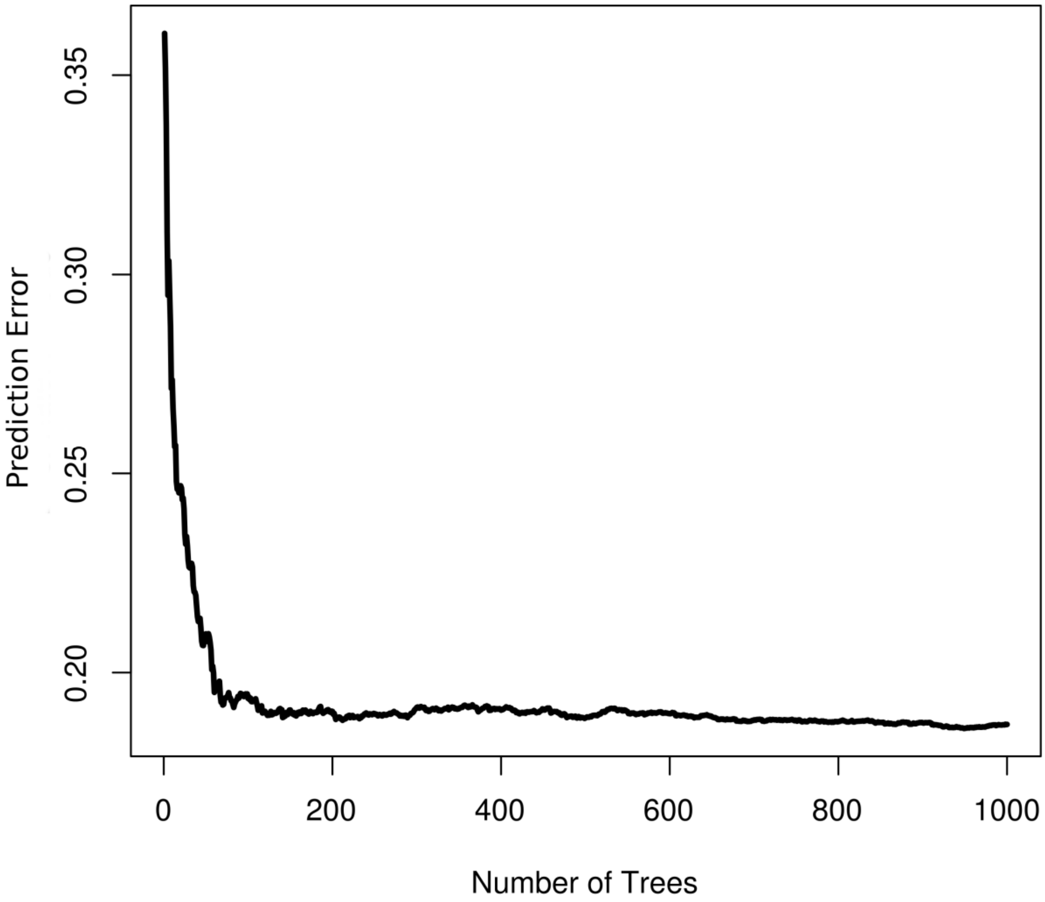

Figure S2: Effect that the number of decision trees included in a random survival forest (x-axis) has in the prediction error (y-axis) of a rheumatoid arthritis mortality model using the log-rank score splitting rule (M_{LRS}).

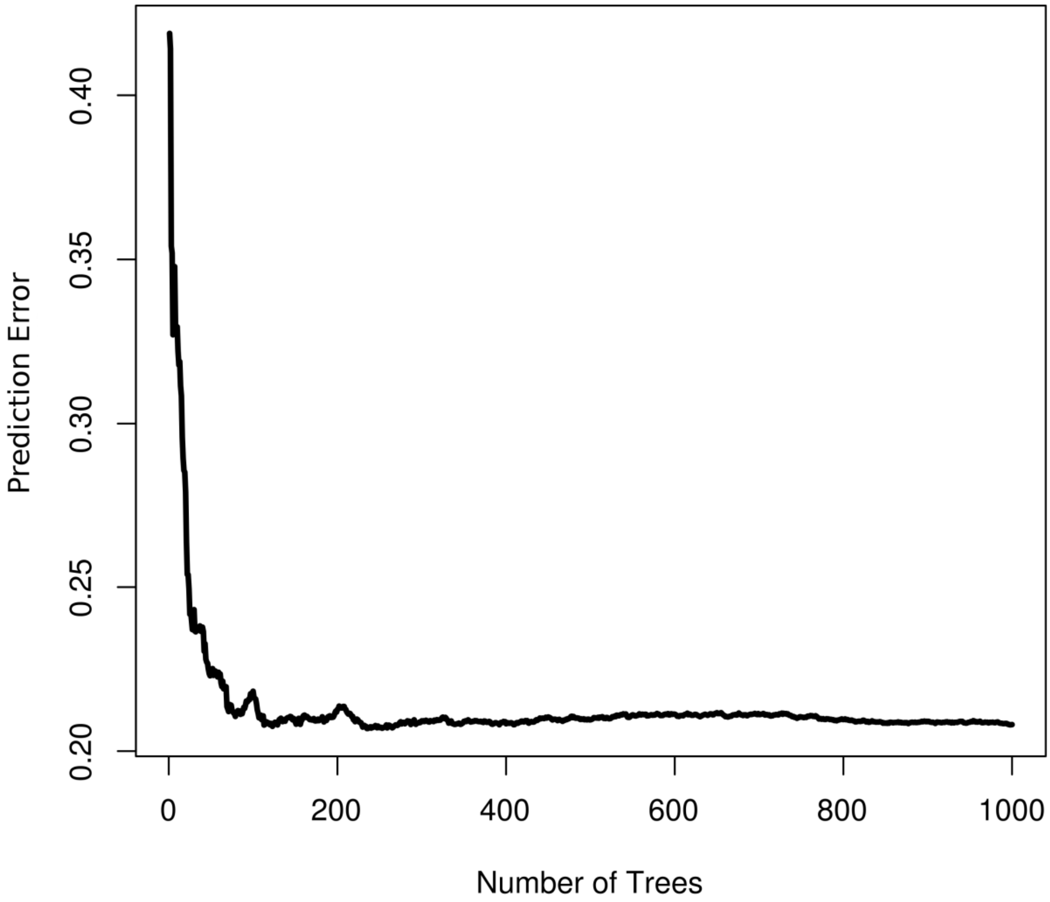

Figure S3: Increase of the integrated Brier score (y-axis) with the follow-up time (x-axis) for two rheumatoid arthritis mortality random survival forest models using either the log-rank (M_{LR}) of the log-rank score (M_{LRS}) splitting rules.

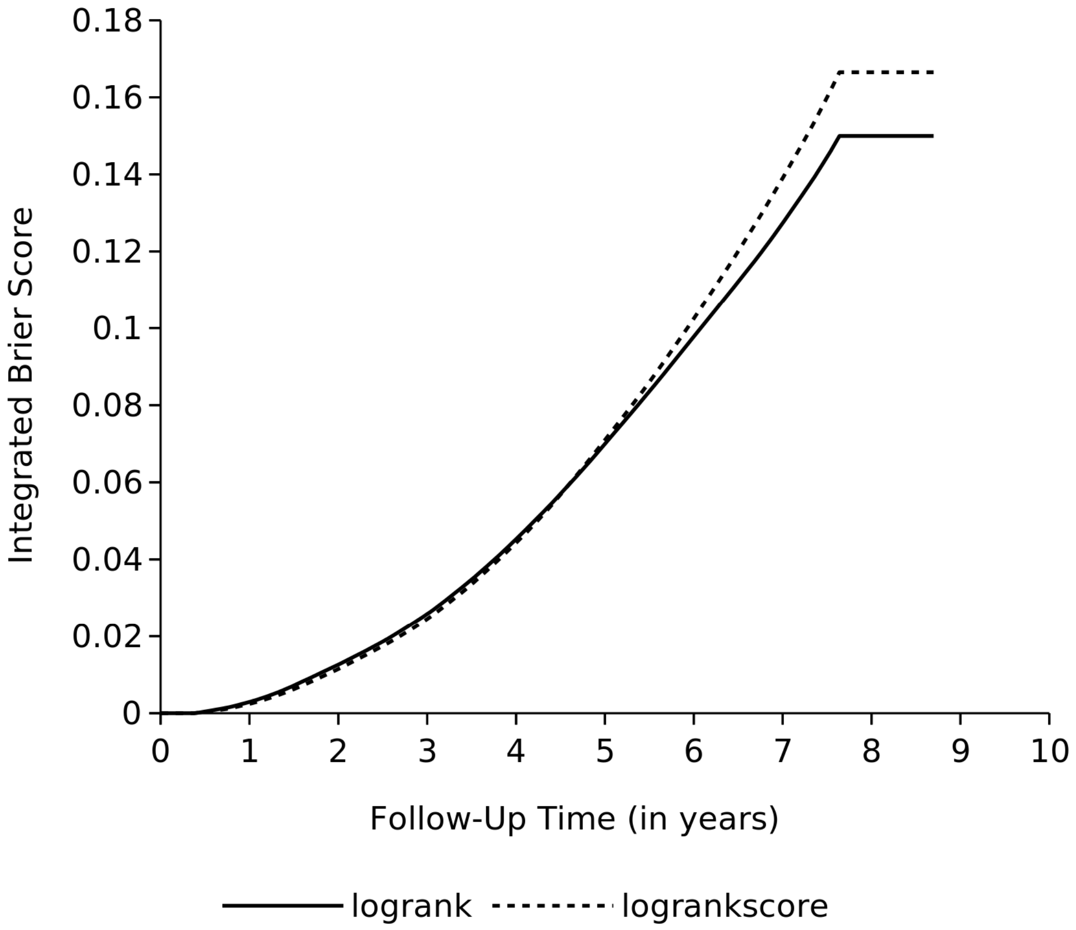

Figure S4: Partial plots representing the adjusted ensemble mortality (y-axis) for each of the variables included in a rheumatoid arthritis (RA) mortality random survival forest model using the log-rank splitting rule (M_{LR}): A. Age at rheumatoid arthritis (RA) diagnosis (in years, continuous variable); B. Median value of the erythrocyte sedimentation rate during the first two years after disease diagnosis (in mm/h, continuous variable); C. Number of hospital admissions regardless the cause during the first two years after disease diagnosis (continuous variable); D. Calendar year at RA diagnosis (continuous variable); E. Spaniard (dichotomous variable; no: 0, yes 1); F. Elapsed time from RA symptoms onset to diagnosis (in years, continuous variable); G. Gender (dichotomous variable; men: 0, women 1); H. Presence of rheumatoid factor (dichotomous variable; no: 0, yes 1); I. Use of biological therapy during the first two years after disease diagnosis (dichotomous variable; no: 0, yes 1). For dichotomic variables, a box-and-whiskers plot with median and percentiles 0.25 and 0.75 are represented. For continuous variables, grey points and black thick dashed lines indicate partial values, and grey thin dashed lines indicate an error bar of two standard errors.

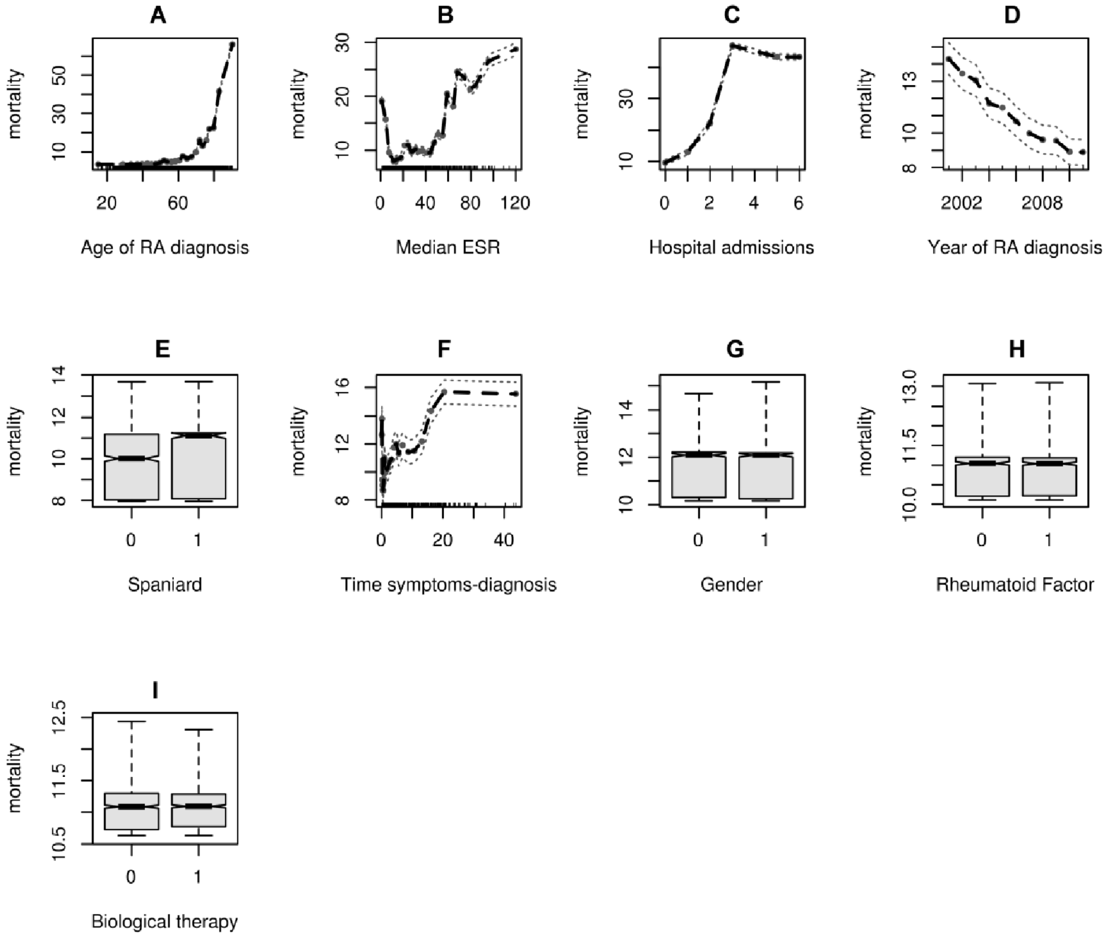
A
mortality
Age of RA diagnosis
B
mortality
Median ESR
C
mortality
Hospital admissions
D
mortality
Year of RA diagnosis
E
mortality
Spaniard
F
mortality
Time symptoms-diagnosis
G
mortality
Gender
H
mortality
Rheumatoid Factor
I
mortality
Biological therapy

Figure S5: Predicted survival curves based on the demographic and clinical-related characteristics of two fictional rheumatoid arthritis patients using a mortality prediction models developed with random survival forest.

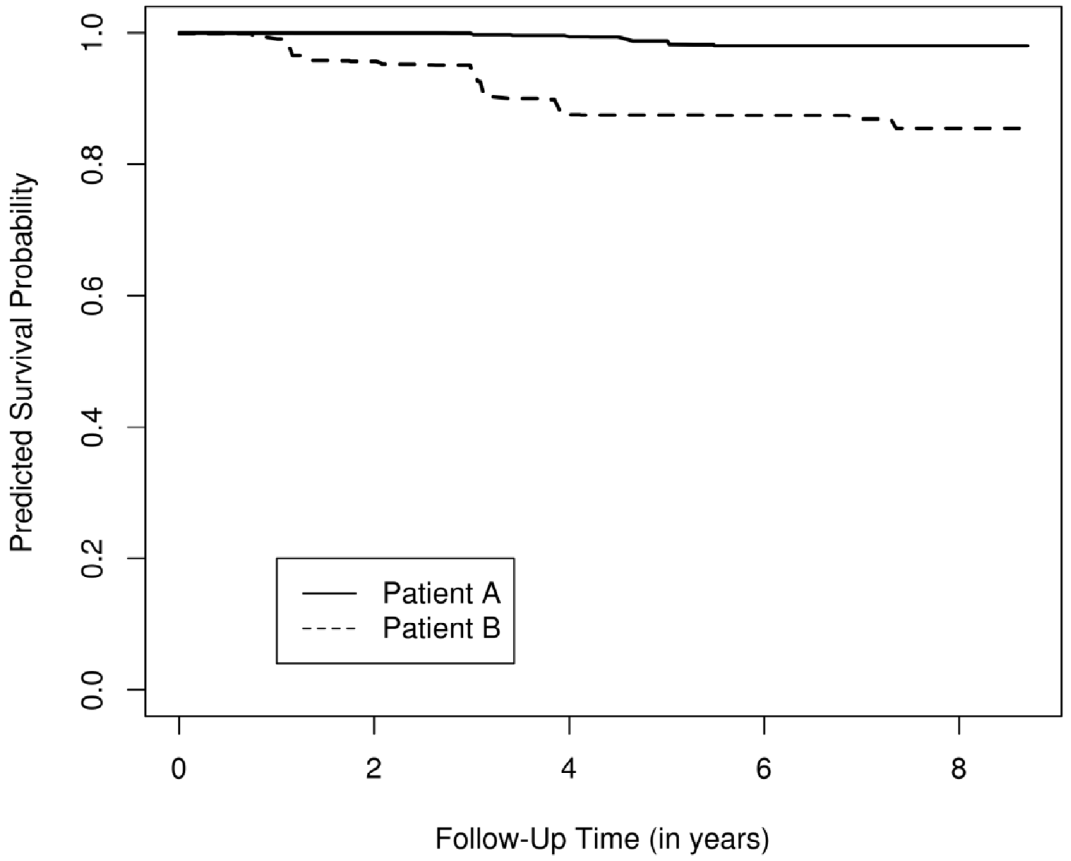

Figure S6: Influence of the predicted ensemble mortality value in sensitivity and specificity, after 1 year of follow-up (starting two years after RA diagnosis), in the HCSC-RAC and in the PEARL study.

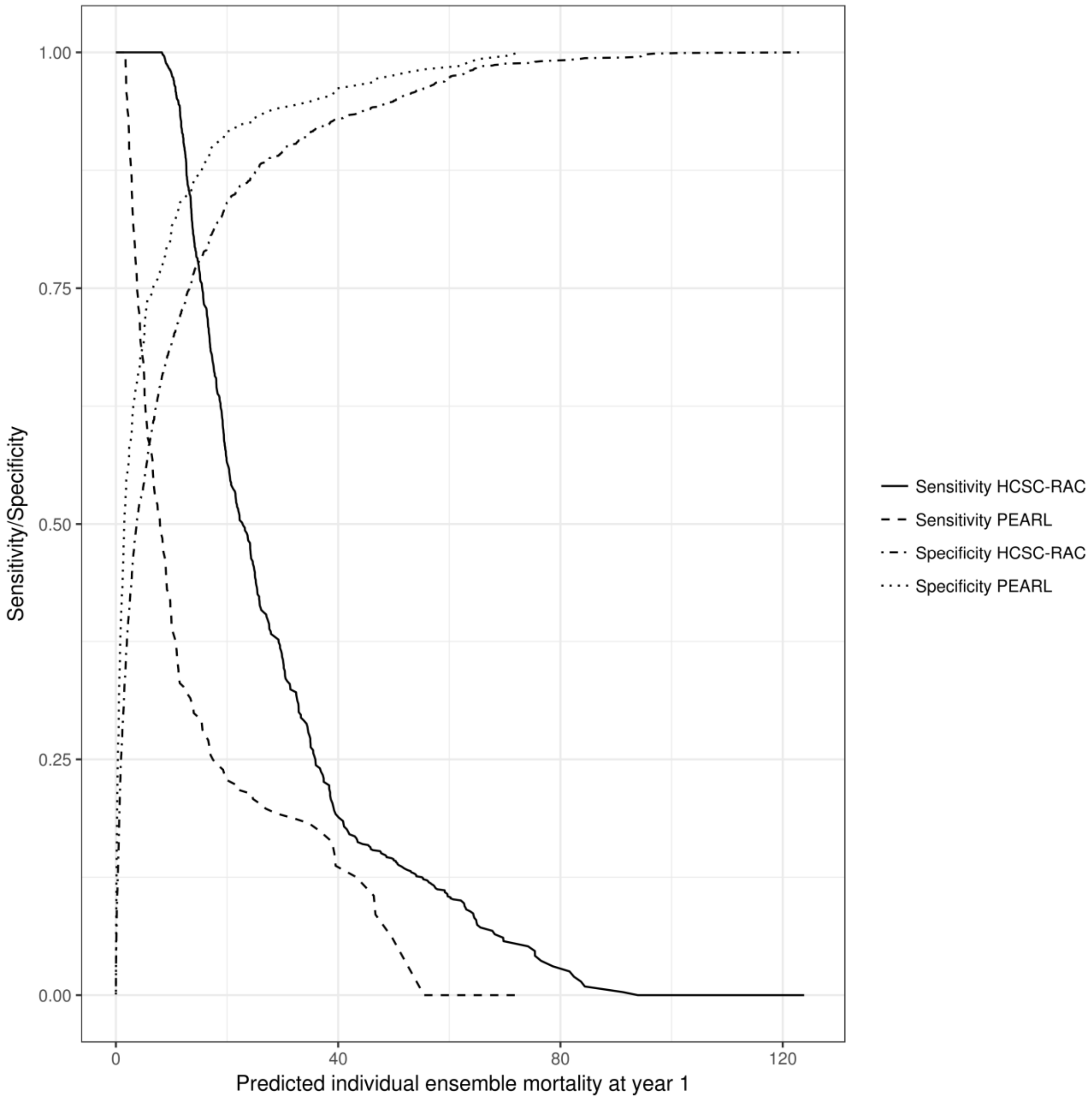

Figure S7: Influence of the predicted ensemble mortality value in sensitivity and specificity, after 2 years of follow-up (starting two years after RA diagnosis), in the HCSC-RAC and in the PEARL study.

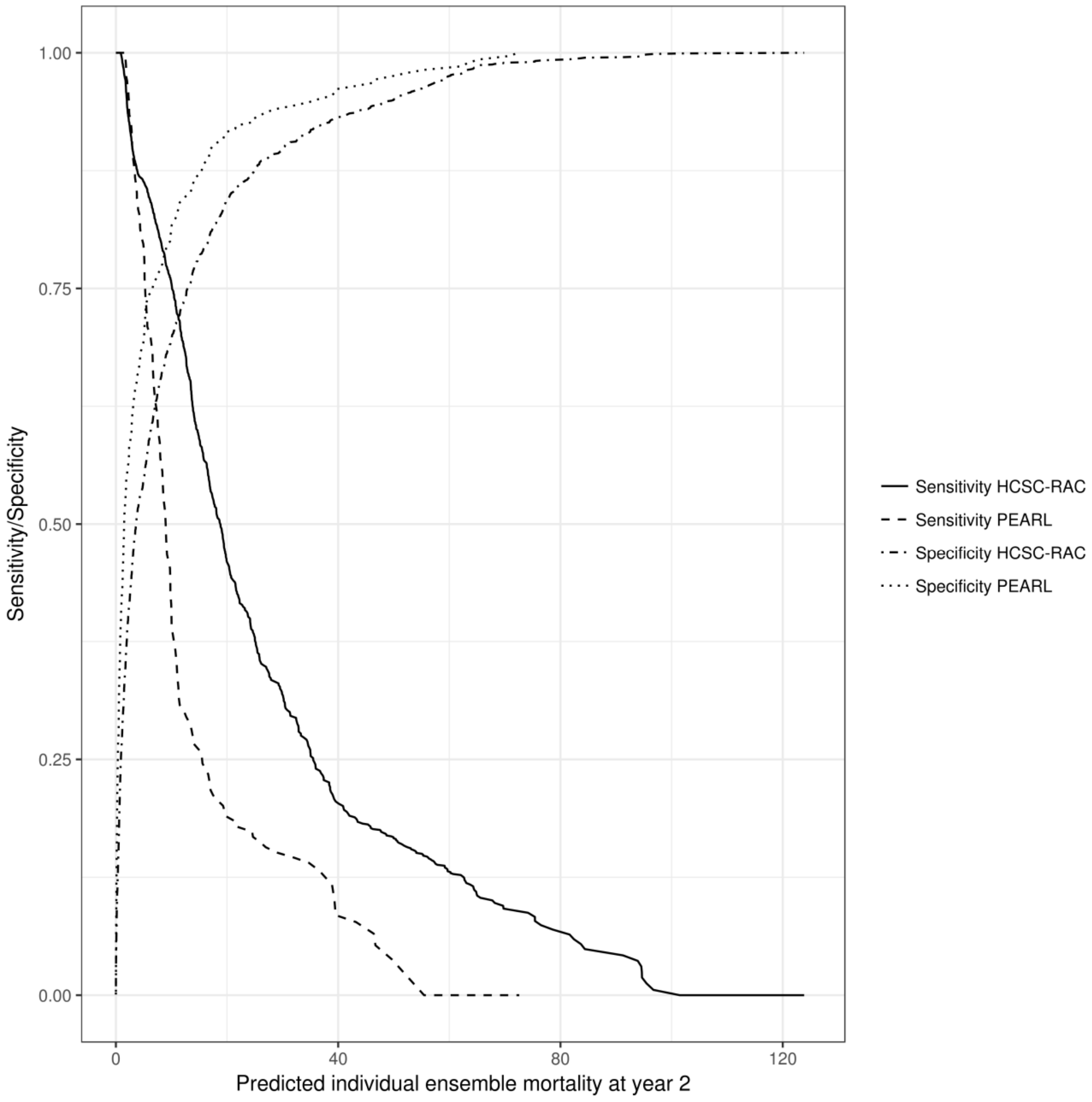

Figure S8: Influence of the predicted ensemble mortality value in sensitivity and specificity, after 5 years of follow-up (starting two years after RA diagnosis), in the HCSC-RAC and in the PEARL study.

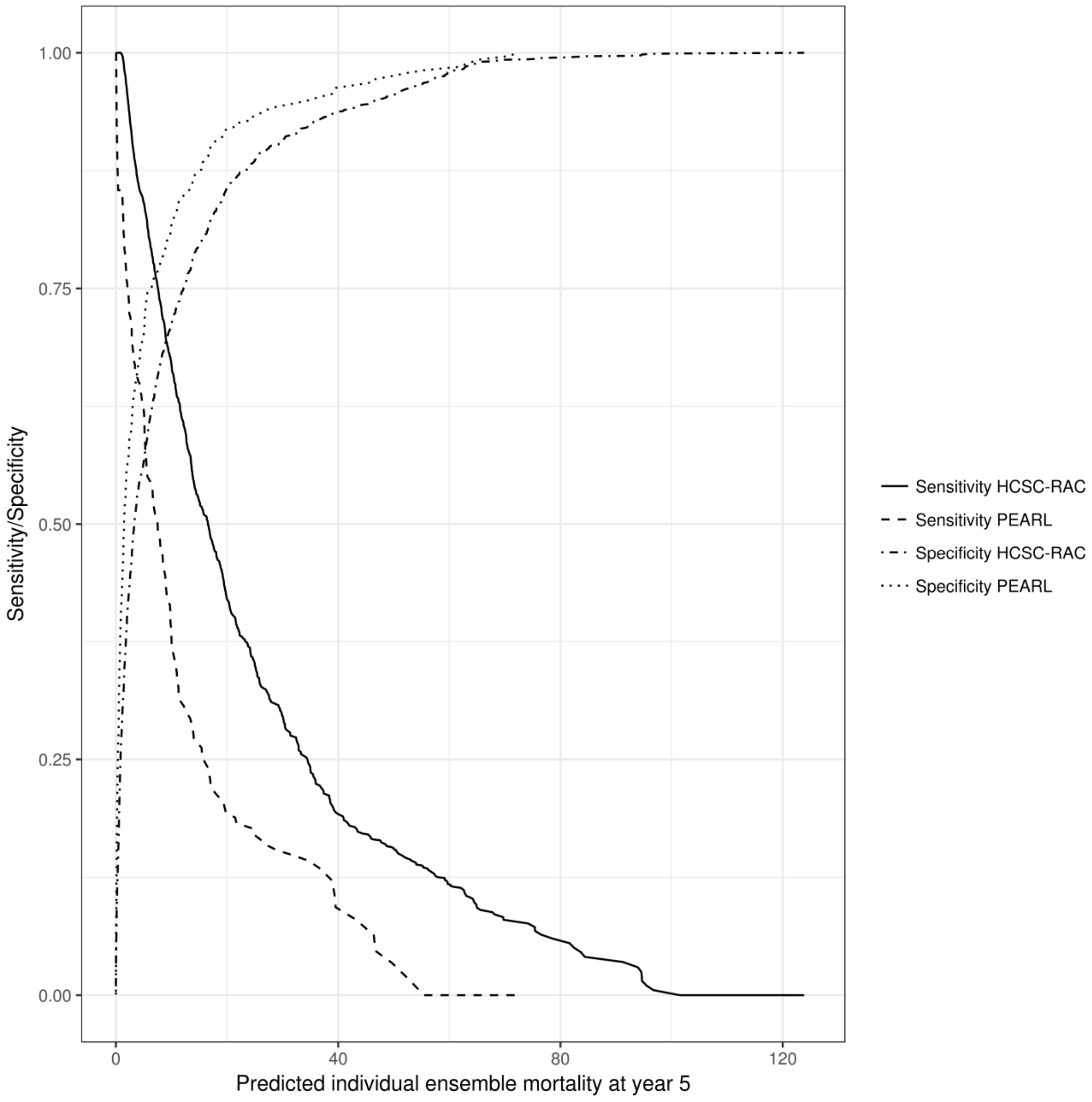

Figure S9: Influence of the predicted ensemble mortality value in sensitivity and specificity, after 7 years of follow-up (starting two years after RA diagnosis), in the HCSC-RAC and in the PEARL study.

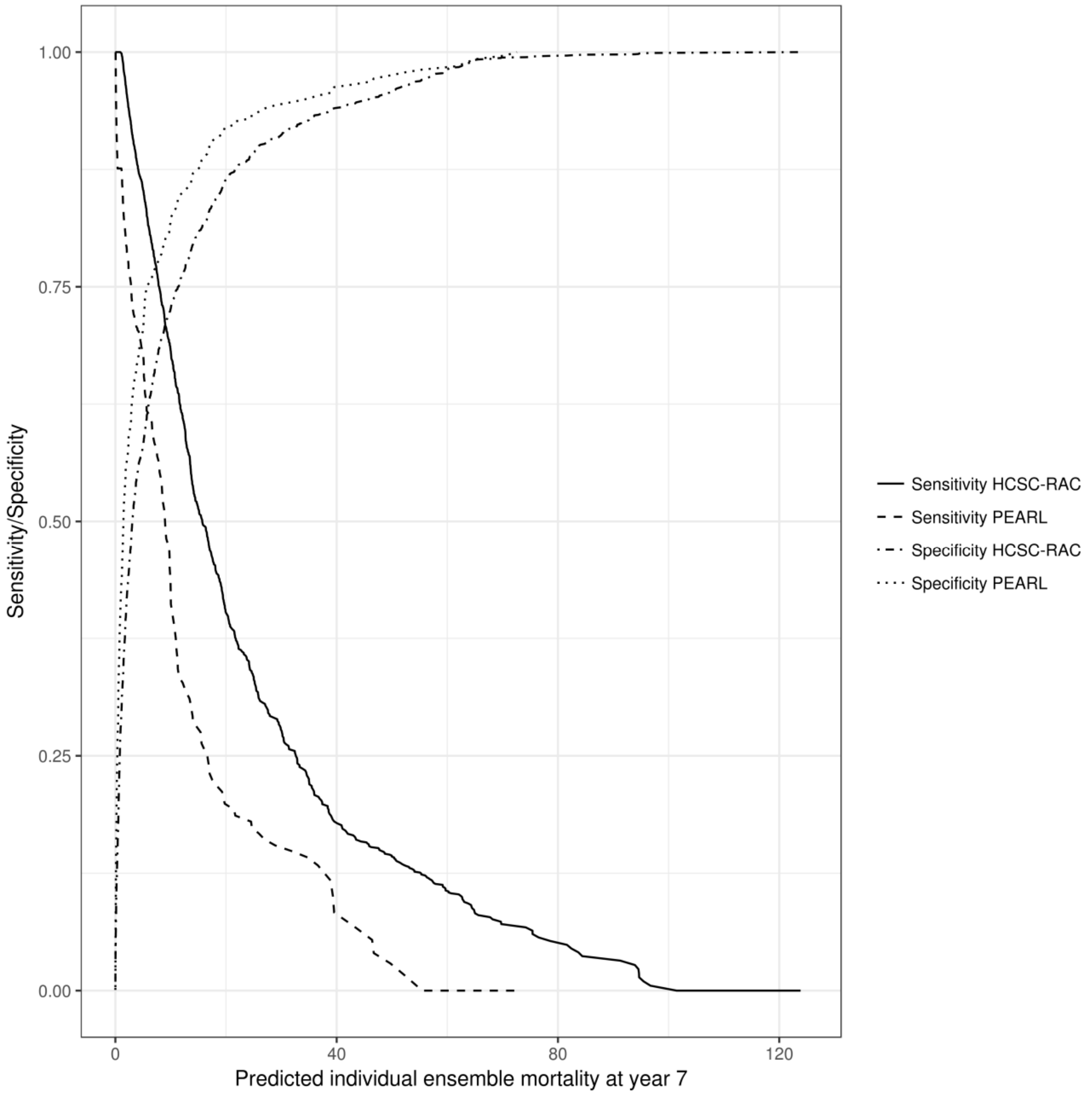

Figure S10: Calibration curves for the M_{LR} in the HCSC-RAC, at 2, 5, and 7 years of follow-up (starting two years after RA diagnosis; continuous line, dashed line and dotted line, respectively).

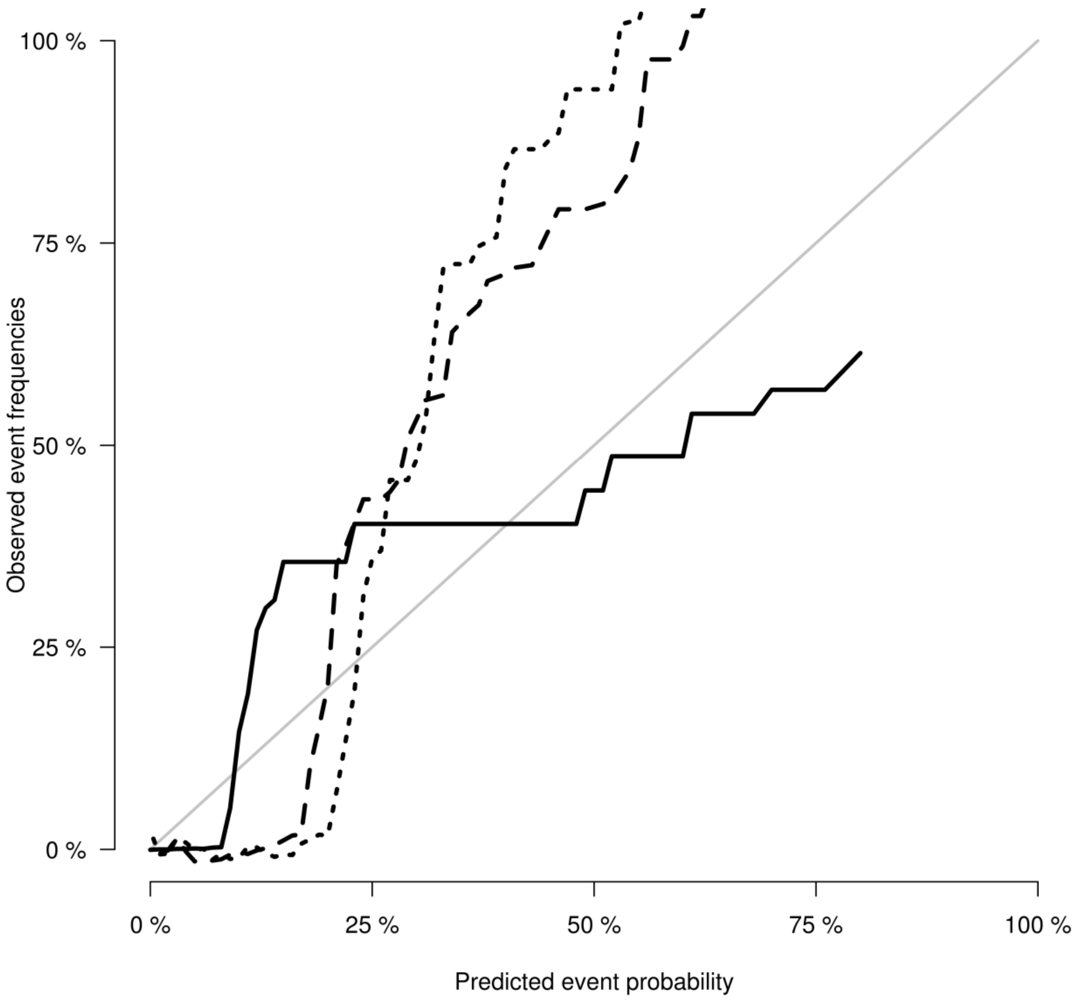

Figure S11: Calibration curves for the M_{LR} in the PEARL study, at 2, 5, and 7 years of follow-up (starting two years after RA diagnosis; continuous line, dashed line and dotted line, respectively).

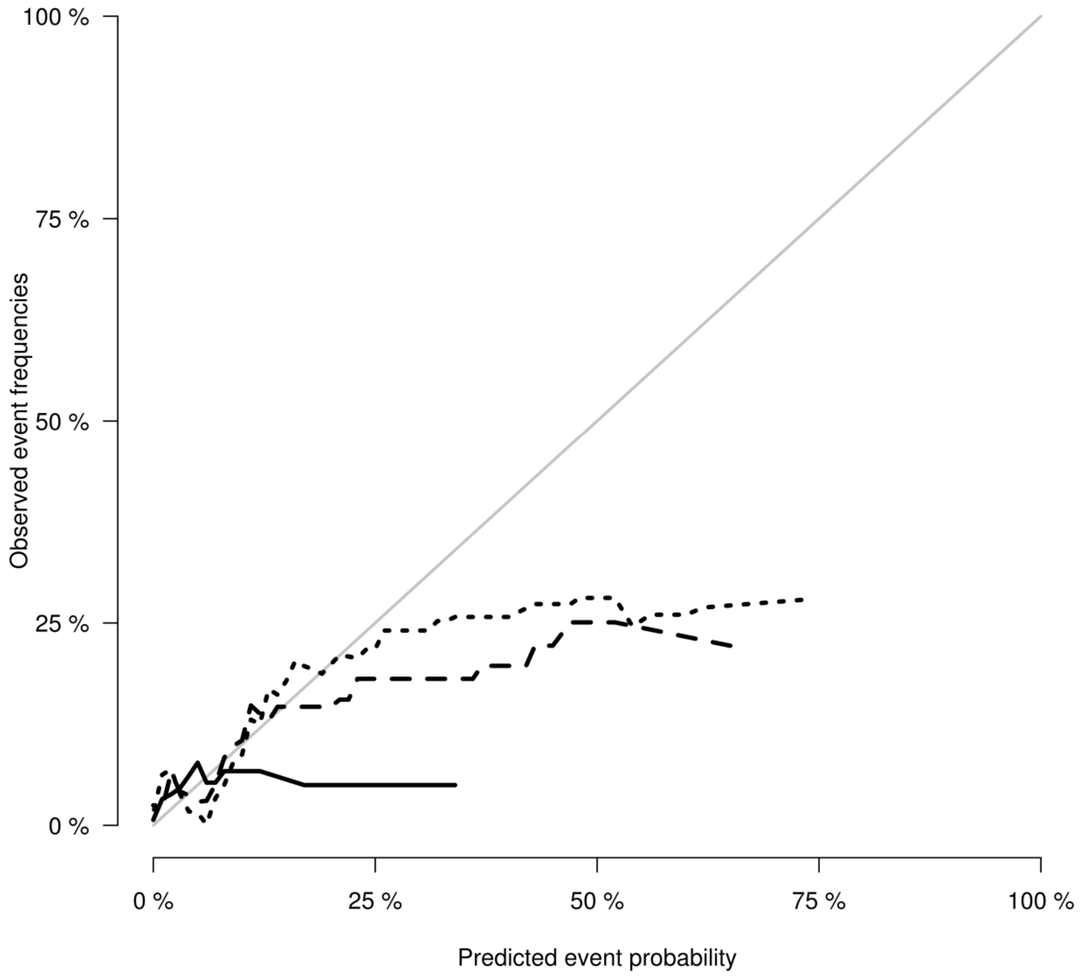

Supplement: Supplementary file 1 — Supplementary Information [file 41598_2017_10558_MOESM1_ESM.pdf]
